# Recurrent Huge Left Bartholin's Gland Abscess for One Year in a Teenager

**DOI:** 10.1155/2017/9151868

**Published:** 2017-10-22

**Authors:** Athanase Lilungulu, Bonaventura C. T. Mpondo, Abdallah Mlwati, Dismas Matovelo, Albert Kihunrwa, Balthazar Gumodoka

**Affiliations:** ^1^Department of Obstetrics and Gynaecology, University of Dodoma, College of Health Sciences, P.O. Box 395, Dodoma, Tanzania; ^2^Department of Internal Medicine, University of Dodoma, College of Health Sciences, P.O. Box 395, Dodoma, Tanzania; ^3^Department of Obstetrics and Gynaecology, Catholic University of Health and Allied Sciences, P.O. Box 1464, Mwanza, Tanzania

## Abstract

Bartholin's gland abscess is the commonest worldwide reported abscess in gynaecological outpatient clinics; it has also been reported that Bartholin's gland abscess is three times more common in occurrences compared to Bartholin's gland cyst. It is more common in women who are at risk of acquiring sexually transmitted infections; however, other causes of infection should be investigated to exclude other causes of disease. We present the case of an 18-year-old female patient, a teenager of the reproductive age group, with the recurrent development of huge Bartholin's gland abscess in a period of one year. The marsupialization surgical technique of repair was performed successfully. She was discharged home and she was scheduled to visit STI's clinic where she was receiving regular screening for STI's and she was also given health education regarding preventive measures for STI's.

## 1. Introduction

Recurrent Bartholin's gland abscess among women of reproductive age is commonly associated with the risk of being in contact with the sexually transmitted polymicrobial infection.

The pathogenesis of Bartholin's gland abscess starts slowly as the progressive swelling of the labia majora which later becomes painful, and finally it is accompanied by fever and massive swelling of the genital vulva on the affected side [1].

The risk of acquiring STIs is related to getting another associated genital tract infection. It is estimated that, among individuals who contract STIs, some of them would likely develop Bartholin's gland abscess, and there is a high chance of acquiring other sexually transmitted infections [2].

It has been reported that Gram-negative bacteria was more commonly isolated than Gram-positive species that are found in the cultivated Bartholin's gland abscess pus; however, the controversy has been observed among directly isolated suspicious species because the abscess is caused by multiple microorganisms [3].

In the pathogenesis of Bartholin's gland abscess, directly induced inflammatory response caused by multiple microorganisms has been associated with the increased risk of acquiring other STIs including HIV and syphilis among the affected individuals [4].

There is high risk of acquiring sexually transmitted infection among individuals with multiple sexual patners and those who practice unprotected sexual intercourse which could be due to decreased body's resistance of clearing the infection and infected individuals would develop persistent infection, which is the risk factor for recurrent sexually transmitted infections [5].

Bartholin's gland is usually associated with secretion of normal presexual intercourse vaginal fluids, and rarely the gland is associated with infection, but it has the possibility of developing Bartholin's gland cyst that can progress to an increased large-sized gland [6]. However, the modalities of treatment by surgical intervention for both conditions remain the same.

We report the case of an 18-year-old female teenager of the reproductive age group with a one-year recurrent huge Bartholin's gland abscess whereby the marsupialization surgical technique of repair was successfully done.

## 2. Case Presentation

An 18-year-old female presented to the gynaecology clinic at Bugando Medical Centre, Mwanza, Tanzania, with a history of recurrent painful huge genital swelling in her left labia majora for one year which initially started as a small swelling, then increased in size, and became painful. She gave a history of pus discharge, and due to its recurrent and persistence swelling, it was associated with fever and inability to walk properly and was accompanied with painful micturition. She had a history of being treated unsuccessfully several times through suction with a needle syringe though she noted a temporary relief. However, she had a history of long-term use of antibiotics without getting relief. The swelling recurred at intervals of less than one or two months over the last year. She has no history of receiving counselling for HIV and testing.

**Figure 1:**
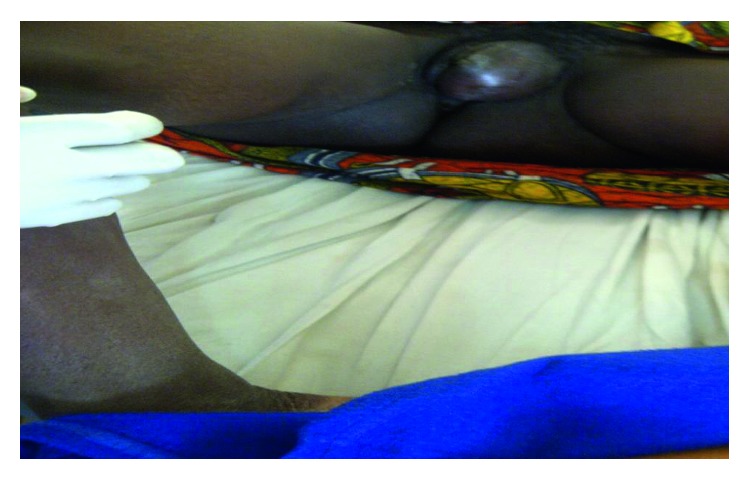
Huge inflamed Bartholin's gland abscess involving the left labia majora and minora presented with shiny smooth surface, discharging pus at the small sinus, with erythematous, fluctuant, tender mass, measuring approximately of 10 × 5 cm.

On examination, there was tender large mass involving the left labia majora and minora, shiny and with a smooth surface, discharging pus at the small sinus, erythematous, fluctuant, and measuring approximately 10 cm in length and 6 cm in width. Her vaginal examination revealed normal vaginal wall, no any offensive discharge, with the healthy cervix; she had negative cervix excitation test and normal sized uterus; neither adnexal mass nor tenderness was elicited (Figure 1()). She had a working diagnosis of huge left Bartholin's gland abscess. On general examination, she had no peripheral lymph node enlargement. Her vital signs were a blood pressure of 120/70 mmHg, pulse rate of 88 beats per minute, and temperature of 37 Celsius. Other systemic examination was normal. Cardiovascular system examination revealed audible first and second heart sounds and no murmurs. Respiratory system examination showed a respiratory rate of 21 breaths/minute, normal chest contour, trachea centrally located, normal chest expansion, and normal breath sound on auscultation. Per abdominal examination, abdomen was flat, moving normally with respiration, and there were no visible superficial veins. The abdomen was soft and nontender. The liver, spleen, and kidneys were not palpable. No shifting dullness and bowel sounds were normal on auscultation.

The patient was counselled for emergency marsupialization, and informed consent was obtained. Preoperative antibiotics were given: ciprofloxacin 500 mg orally 12 hourly for 5 days, metronidazole 400 mg orally 8 hourly for 7 days, and doxycycilline 100 mg orally bid for 7 days. In theatre, surgical intervention by marsupialization was done under spinal anaesthesia (5% lidocaine 50 mg preparation). The patient was placed in the lithotomy position, and left huge Bartholin's gland abscess was exposed. The incision was made through the gaped skin covering the abscess whereby pus was drained and cleaned with normal saline until the underlying fresh tissue edge of a gland was identified and was oozing fresh blood. A lot of foul-smelling pus was drained from the abscess, approximately 30 ml. The edges of the gland were grasped gently using a forceps. The sutures material used was a vicryl number 2-0, and repair by the marsupialization method was done. In the postoperative care, she continued with already prescribed antibiotics, and the analgesia given was paracetamol 1 g orally 8 hourly for 3 days. The patient was discharged on the 3rd day and linked to reproductive health clinics for counselling about the knowledge of sexually transmitted infection prevention and treatments.

## 3. Discussion

In our case, the patient presented with a history of recurrent left huge labial swelling for the past one year. The reason of recurrence probably was suggested to be the previous treatment by incision and drainage rather than treatment by marsupialization-type incision and followed by unknown prolonged course of oral antibiotics [7].

The microorganisms causing recurrent Bartholin's gland abscess are polymicrobial and often commensal microorganisms that are not sexually transmitted. In our case, the likely hood that, there was high chance the Batholin's gland abscess was caused by a sexually transmitted infection was the fact most teenagers are sexually active [8].

Treatment of Bartholin's gland abscesses depends on the presenting symptoms that may indicate the cause of that abscess; however, if it happens that it started to present asymptomatically, it may require marsupialization only without issuing the polymicrobial antibiotic treatment [9].

Bartholin's gland cysts and abscesses may present with different symptoms, and the required surgical management should be marsupialization and not incision and drainage. Though the incision and drainage procedure was shown to be relatively quick and easy to perform and was proven to be quick on cure rate, among patients receiving this type of surgical technique, it has been shown to have increased tendency of recurrences in a patient with Bartholin's gland abscess [10].

The use of systemic broad-spectrum antibiotics in this patient aimed to cover polymicrobial species of bacteria. However, it has been reported that some of the bacteria isolated from Bartholin's gland abscess are normal vaginal flora in origin, and therefore prescribing antibiotics increases the risk of infections in susceptible immuno-incompetent individuals [11].

In our case, there was a history of recurrent Bartholin's gland abscess, and this might be due to the repeated exposures to the infections or improper use of strong broad-spectrum antibiotics together with incision and drainage instead of management by marsupialization, as we know that incision and drainage have been shown to increase risk of Bartholin's gland abscess recurrences [12].

Having a huge Bartholin's gland abscess as the case in this patient and prescribing multiple antimicrobial agents with incision and drainage alone may generally not solve the issue of recurrent Bartholin's gland abscess. The marsupialization surgical technique done in this patient has been shown to be a successful surgical repair, on the progressive follow-up of the patient through the gynaecological clinic. However, additional use of broad-spectrum antibiotics has also shown to support the improvement of the recurrent Bartholin's gland infection [13].

## 4. Conclusion

Bartholin's gland abscess should be distinguished from other vulvar masses. A simple management by marsupialization and broad-spectrum antibiotics has been proved to be effective rather than management by surgical incision and drainage alone.
